# Patterns and Facilitators for the Promotion of Glaucoma Medication Adherence—A Qualitative Study

**DOI:** 10.3390/healthcare9040426

**Published:** 2021-04-07

**Authors:** Stefanie Frech, Rudolf F. Guthoff, Amin Gamael, Christian Helbig, Annette Diener, Manuela Ritzke, Anja Wollny, Attila Altiner

**Affiliations:** 1 Department of Ophthalmology, Rostock University Medical Center, Doberaner Str. 140, 18057 Rostock, Germany; rudolf.guthoff@med.uni-rostock.de; 2 Ophthalmic Care Unit, Rostock University Medical Center, Doberaner Str. 142, 18057 Rostock, Germany; Amin.Gamael@umr-mvz.de; 3 Institute of General Practice, Rostock University Medical Center, Doberaner Str. 142, 18057 Rostock, Germany; Christian.Helbig@med.uni-rostock.de (C.H.); annette.diener@web.de (A.D.); Manuela.Ritzke@med.uni-rostock.de (M.R.); Anja.Wollny@med.uni-rostock.de (A.W.); altiner@med.uni-rostock.de (A.A.)

**Keywords:** primary open-angle glaucoma, adherence, qualitative study

## Abstract

Primary open-angle glaucoma (POAG) is a chronic optic neuropathy causing irreversible nerve fiber damage. Initially asymptomatic, it progresses slowly without any notable sign of vision loss, thus early detection and treatment is essential. The standard treatment being non-invasive topical administration of eye drops harbors the problem of patients not being adherent. This study aimed to explore the experiences and thoughts of glaucoma patients about their medication management to improve our knowledge on how adherence works for the individual patient. Narrative interviews were conducted with 21 glaucoma patients. Data were analyzed using inductive content analysis and the documentary method. Different patterns of adherence were identified which depended on personal biographies, living conditions, or the patient’s knowledge about the disease. Interpreting eye drop medication as a ritual, a task, or routine was helpful for some patients, whereas other patients, who were aware of the consequences of not taking the drops, were motivated by intrinsic or extrinsic factors. The patterns identified here represent strategies for managing and adhering to daily glaucoma medication at an individual level. Linking daily drop application or medication intake to these patterns may help to promote correct medication management of patients with chronic diseases who lack a regular medication regimen.

## 1. Introduction

Glaucoma, an optic neuropathy, is characterized by the damage of the optic nerve. Disease progression can result in severe visual impairment and vision loss. Medical and surgical therapy focuses on the decrease of intraocular pressure as the main risk factor [1]. The lifelong application of eye drops, which sometimes have to be applied several times a day in order to slow progression, may be associated with inconvenience and uneasiness for the patients. The main reason for a decrease of treatment effectiveness or failing of medical therapy is lack of adherence to the eye drop regimen. Adherence is a complex phenomenon, influenced by multiple factors such as self-administration, side effects, level of education, forgetfulness, and prescription filling [2,3,4,5,6,7]. Additional factors related to adherence involve treatment-, environmental- and provider-related influences [8].

In this qualitative research study, narrative interviews were used to learn from glaucoma patients about their glaucoma management and their medication adherence patterns with the intent to interpret individual cases for the formulation of potential general findings about medication management. A characteristic of qualitative methods is to address issues which are difficult to be clarified with quantitative methods, e.g., examination of personal views and individual cases. Interviewing patients gives detailed insights into their perspectives and views of given circumstances. Furthermore, the face-to-face situation enables the interviewer to ask for more detailed information by re-casting questions using specific key words mentioned by the patient. Finally, in qualitative research, patients’ attitudes, beliefs, and preferences and the question of how evidence is turned into practice are investigated [9]. In summary, motivations and reasons underlying certain behaviors and the reasons for these can be revealed.

Recent studies employing interviews with glaucoma patients focused on reasons for non-adherence [10,11,12]. Our study used a different approach, namely to ask the question of how adherence works for the individual patient. In the first place, we aimed to explore and to learn about strategies, experiences, and thoughts of patients related to their individual daily drop application management, rather than identifying more factors related to non-adherence. Identifying patterns of glaucoma medication management of patients will help to increase our knowledge on adherence and individual strategies of medication management. Identifying such patterns might make non-adherent patients more sensitized to the issues; they can profit from the knowledge of adherent patients and positively influence their own adherence adapted to their own living conditions. Adherence works on an individual level and patients differ in their understanding of the disease. An individual teaching strategy is necessary to increase the awareness of why a daily and regular medication management is of utmost importance. As a consequence, the identified patterns could be integrated into individual daily routines and living habits of patients to increase adherence, leading to improved regular medication management. In addition, these patterns might potentially be relevant for the management of medication of other chronic diseases, such as diabetes or hypertension [13,14].

## 2. Materials and Methods

### 2.1. Design and Setting

For this study, 21 glaucoma patients were interviewed face-to-face using the narrative interviewing technique. Patients were recruited randomly from the Department of Ophthalmology of the Rostock University Medical Centre where they stayed for a 24 h intraocular pressure (IOP) analysis or from the Outpatient Unit of the Rostock University Medical Centre.

Inclusion criteria were the diagnosis of primary open-angle glaucoma according to ICD-10 code H40.1 and the application of daily eye drop medication for at least one year. No data on patients’ adherence were collected prior to the study, these were obtained from the patients’ answers during the interview. Interviews were conducted by trained researchers, and interviews took place in a private room at the recruiting places. The study was approved by the Ethics Committee of the Rostock University Centre (reference number A2016-0156). All methods were performed in accordance with the relevant regulations and guidelines, and written informed consent for inclusion was signed by all patients before participating in the study.

At the beginning of each interview, an openly formulated narrative stimulus was given: “When was your glaucoma diagnosed and how do you experience it in your daily life? Please tell me everything that comes to mind.” This introduction was chosen to encourage the patient to talk about the disease and to gain insights into the course of the disease. In addition, the interview guide covered different open-ended questioning sections, such as the disease itself, doctors’ visits, eye drop management, and situations which led to non-adherence, and recommendations for other patients, unless these had already been provided by the patient. To ensure that everything important was reported, the interviewer invited the patients to deepen or contribute further to the material discussed at the end of the interview (Table 1). In total, 417 min of recording were analyzed, pseudonymized, and transcribed verbatim using the transcription software f4transcript (Audiotranskription, Marburg, Germany). Analysis was conducted by an interdisciplinary team of physicians and social- and natural scientists.

Data analysis was performed using inductive content analysis and the documentary method with comparative analysis and typification. The method of inductive content analysis was used to identify themes and topics of the interviews by repeated examination and comparison of the data without initially relating it to the aim of the study. The documentary method was applied to reconstruct experiences from the patients and to interpret the content of the interviews. This method allows a more in-depth analysis, beyond mere description, and relies on a consistent comparative analysis. It archives its empirical results in forms of types that allow a generalization of the results [15,16]. The analysis group agreed that data saturation was reached after 16 interviews. At the point where no new or additional insights into the topic are gained through additional interviews, data saturation was reached, which was confirmed with the analysis of the 5 remaining interviews.

The interpretation of the interviews and the analysis of the results refer to a rational connection between an experience or an action and the intention behind an action. The method of type formation, which was used for analysis, involves abstracting the experiences of one patient and comparing these with experiences of others in a typifying way. If an experience can be distinguished from another experience and if it refers to commonalities of the cases, then it can be detached from an individual level and elaborated to a type. Comparative analysis serves type formation, thus it is applied throughout the entire interpretation process with the goal of finding patterns independent of the individual to archive generalizable statements [15,16].

### 2.2. Sample Characteristics

The mean age was 73.1 ± 8.9 years with 12 female and 9 male patients. Of the 21 patients, 4 were employed and 17 were retired. Table 2 depicts the age and sex of the participants.

## 3. Results

**Figure 1:**
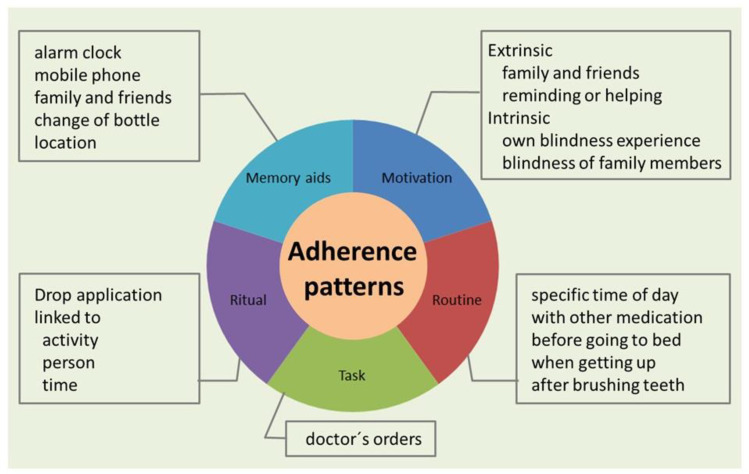
Identified patterns of adherence. The patterns are linked to specific actions or behaviors of people, which helps them to apply eye drop medication regularly.

In the course of the interviews, patients provided detailed information about personal adherence habits as part of their medication management. A number of recurring patterns were revealed which seemed to influence adherence positively, providing a certain self-confidence for the patients about their drug management. Some followed a strict routine of drop application, some were motivated by personal experiences. For others, drop application was a kind of ritual coupled with other activities (e.g., persons or time). Other strategies of thinking about when to take the drops were reminders. For some patients, simply the ophthalmologist’s request to remember to take their drops regularly was motivation enough. Others were motivated by the fear of going blind or by experiencing blindness in their own family. These various patterns are summarized in Figure 1 and described below in more detail using quotations from the interviews.

### 3.1. Motivation

The motivation to think about regular drop management and the subsequent action of drop application was coupled with different patterns. Motivation can either be intrinsically based or triggered by extrinsic factors. Different patterns were evident for both types of motivation.

#### 3.1.1. Extrinsic Motivation

This type of motivation was triggered by family members, mainly by the spouse, or by friends. Extrinsic motivation was either provided by reminding patients to take their drops, or by providing help with applying drops because of a physical limitation of the patient. This pattern of adherence was often performed out of habit, and had been successfully established for years. Either way, drop management was taken over by another person, someone other than the patient himself.

Quotations:

“And we do it very conscientiously and every evening at half past nine it’s my time to take my drops and my wife is doing this well. So this puts me in a stable situation”

(PDA09)

“And when I’m out with a group of friends, I get a phone call, then someone from the group gives me my drops. Yes, my husband calls me to say: eye drops!”

(PDA18)

#### 3.1.2. Intrinsic Motivation

The main intrinsic factor of motivation to take drops regularly was the fear of going blind triggered by either blindness of family members, like a mother or a cousins’ son, or by a person’s own experiences with episodes of blindness. Some patients had had unusual experiences of vision loss after surgery or had already lost sight in one eye due to glaucoma. Adherence was therefore a positive motivator which contributed to stable eyesight, increased by the fear of blindness.

Quotation:

“But if you know you have to, and if it’s about keeping your eyesight, then you can do anything”

(PDA17)

“You know, if you only have one eye left, then you´re gonna get, how should I put it, you get scared, you know?”

(PDA15)

### 3.2. Ritual

Linking drop application to another activity, to a specific location or time, or involving another person, provided patients with patterns that helped them remember to apply the drops regularly. Some patients even combined activities, such as time and person or activity and person. As a consequence, applying drops became a ritual.

Rituals are of symbolic character, not only what is directly be done or said, but also hidden meanings are conveyed. The affinity to a ritual is emotional and affective [17]. Rituals are by their nature reflective of a shared understanding about how to behave and are clearly located within a social context. They also serve as devices that reduce anxiety arising from uncertainty about what to do in certain situations [18].

For some patterns it seemed difficult to distinguish between routine and ritual, as there is a whole range of intermediate options for classifications. Here, a ritual was described when combining an action with another activity or another person, setting it apart from a routine.

Quotation:

“When I take my drops, say in the evening, let´s start in the evening. Well, then I’ve already taken the drops and so then I put the bottle on my facial care jar for the next morning. And then, before I can apply my facial care cream, I need to take the bottle of drops off of it”

(PDA06)

“We take the drops at 8 pm, when it’s time for the weather forecast then it’s time for the drops, so we don’t forget them. Yes, so just before the weather forecast comes on, my husband gets up and gets the drops. That’s just part of it now”

(PDA18)

### 3.3. Routine

A routine will become a habit after multiple repetitions. Patients reported that such routines were linked to different actions. Drop application was performed at specific time points, was associated with the intake of other medications, or was linked with going to bed or getting up. Some patients followed these routines very strictly, as a part of their daily life. Older patients described routines as a positive feature to organize their daily lives, as they did not have to think too much about daily sequences. Thinking about applying eye drops is not a problem for them as long as the habit is maintained. If a routine was omitted or changed, then the risk of missing an application seemed to be increased.

Lavie and colleagues talk about routines as a constantly evolving pattern for the implementation of a particular task [19]. They reduce the complexity of decision-making, and make behavior more predictable [18]. According to Black and colleagues a routine can be defined as a repetitive behavior involving a momentary task that requires little conscious thought [20].

Quotation:

“It has become second nature to me, just like brushing my teeth and getting undressed”

(PDA02)

“Man is a creature of habit, right?”

(PD03)

“I swallow my pills, I apply the eye drops”

(PDA13)

### 3.4. Task

For some patients, the daily application of eye drops was a task to be performed because of a doctors’ orders. These patients did not think about forgetting to take the drops, as the strict order from the doctor did not leave much room for this option.

Quotation:

“Well I do as I´m told. I think that’s the right way to do it. Well, that’s how we live”

(PD03)

“I try to take it regularly, as it’s been prescribed, and then that’s the way it is for me”

(PDA12)

### 3.5. Memory Aids

Memory aids included not only appliances, which helped patients to remember to take their drops, but also individuals, mainly family and friends. A simple reminder or inquiry of a trusted person often helped to induce action. Alarm clocks and mobile phones were the main memory aids used. Patients often developed individual processes, such as having numerous alarm clocks nearby, including alarms placed in a purse or pouch if they were going to be out and about. After the patients applied the drops, they would reset the clock to the next time of application. Another memory aid or indicator that eye drops had to be applied was changing the location of the bottle after each drop application.

Quotation:

“Yes, yes, when I sit and read in the evening, then I have it set for about 8. It rings, and the last one at 10, and then it’s automatically set to 8 a.m. and then, well, that´s part of my life, I hardly notice it anymore […] I’m just always armed with an alarm clock”

(PDA17)

## 4. Discussion

This qualitative study was performed to analyze adherence of glaucoma patients in order to identify patterns and facilitators which help to increase long-term adherence. Narrative interviews were conducted to gain insights into the patients´ perspective of glaucoma and their medication management. Several patterns of adherence which were used by glaucoma patients to think about and to remember a daily medication were identified. One of these patterns was fear of blindness. Patients reported either an individual experience, the thought of becoming blind, or the fact that a family member had experienced blindness. The fear of going blind seemed to be a strong motivation to adhere to a daily eye drop application regime.

The assessment of non-adherence of glaucoma patients using qualitative methods was described earlier. One study described motivation to prevent vision loss as a factor [21]. However, fear of blindness is only a facilitator for patients who have already actively thought about the disease and its consequences. Taylor and colleagues found that forgetfulness and a lack of glaucoma education were key factors for non-adherence in the USA. The authors concluded that there was a need to identify motivational factors of patients [10]. In the UK study of Lacey and colleagues, the primary reasons for non-adherence were age and individual differences as well as lack of education, leading the authors to conclude that tailored approaches may improve glaucoma patient’s motivation for regular drop application [11]. In 2010, Stryker and colleagues performed interviews including open-ended questions about medication usage, barriers to medication adherence, medication knowledge, health literacy, and social support with the aim to develop an intervention [12].

Categories of adherent patients had been described by Pound et al., 2005. Patients were deemed to be active acceptors, and they showed a willingness to follow a medication regimen based on an existing understanding of the disease [22], as was also experienced in this study. Knowing about the progression and experiencing blindness or an inclination to follow the doctor’s orders were identified as very strong motivators. In contrast, passive acceptors were those who relinquished control to others [22]. In our study, some patients used memory aids or described their regimen as a routine or a ritual which was partly closely linked to the help of other persons, such as family members or a spouse. Some patients described how others helped them to administer the eye drops, but a few reported that they had even made a complete transfer of responsibility. Routines and rituals are distinguishable in certain points having various implications for individual aspects of health and for the promotion of health and well-being. Routine might be linked to a special time or place; the ritual aspect requires an additional creation of meaning over time [17]. Nevertheless, any routine has also the potential to become a ritual once it moves from an instrumental to a symbolic act [23]. The implementation and support of routines and rituals in healthcare has also been shown for the management of other chronic diseases and family health, e.g., diabetes [17].

Lunnela and colleagues interviewed glaucoma patients and were interested in the attitudes and views on education and social support methods. They concluded that patients were relatively satisfied, nevertheless, new and timesaving methods of education and support should be developed to cope with glaucoma treatment [24]. Waterman and colleagues identified nine categories of health education needs derived from qualitative interviews. One main outcome was that group education was an attractive possibility for patients [25]. Group education would also support the findings of this study, as non-adherent patients could learn about routines and rituals from adherent patients, including, e.g., linking the application of drops with other activities which serve as reminders, as described previously [21]. According to the FDA, sticking to a medication routine means taking medication as prescribed in the right dose, at the right time, in the right way and frequency [26]. As noted above [10], patients can talk about any lack of understanding or confusion about administering eye drops more openly when they are in a group setting. The use of health coaching sessions and telephone coaching for glaucoma care in the USA also revealed an increase adherence to eye drops in response to interventions [27]. A direct question about usefulness was asked in the 2013 UK study of Somner and colleagues who were interested in patients´ thinking about personal health records to address adherence. Multiple focus groups involving a total of 71 participants were conducted and narrative data were collected. Their conclusion was that listening and responding to viewpoints of patients is fundamental [28]. An additional step which would further promote the individualization of interventions to improve glaucoma care and awareness would be to tailor the coaching to individual needs. A tailored behavioral change program was developed by Killeen et al., 2016, with the intent to improve medication adherence of glaucoma patients. They interviewed 21 glaucoma patients and identified important themes for the improvement of self-management, like social support, medication routine and others. In conclusion, patients stated satisfaction with the tailored content of the study [29].

One intrinsic bias or limitation of our study might be that interviews were conducted with those patients who had a doctor’s appointment. It is possible that these patients might be the more adherent ones in general. However, as our goal was to examine adherent behavior, we are convinced that this was the correct group of patients with whom to discuss the issue. Nevertheless, it is possible that they might have given over-positive reports about their own patterns of adherence. Therefore, one might need to be careful when extrapolating and generalizing the results to other glaucoma patients. Furthermore, no data about the number of medications used or the range of stability of glaucoma were collected. Nevertheless, it would be interesting in future studies to correlate patient-specific clinical data with the personal views of patients. The results of this study and the information gained from the patients provide a basis for the development of intervention strategies which might help glaucoma patients to develop an individual medication-taking behavior, gaining a high level of adherence for daily drop application.

As glaucoma demands life-long daily treatment involving a more or less uncomfortable and complex medication management, strategies need to take into account patients’ individual characteristics, and more than one strategy might be necessary [30], e.g., linking pill taking to patients’ daily habits and rituals [31]. Increasing the awareness and the motivation of applying the drops regularly is a major challenge. Healthcare providers and patients might find ways to implement considerations into their lifestyle to increase long-term adherence [32].

The results of this study create a basis to develop potential interventions to improve adherence of patients and to integrate application of eye drops into the daily lives of individual non-adherent patients. Multidimensional interventions, and a comprehensive package of adherence support, improve adherence and patients’ quality of life [21]. Improved progression in pharmacotherapy is important to limit side effects and to find out about combination therapies. Furthermore, micro-invasive glaucoma surgery is an evolving field that can provide patients with the best possible therapeutic options. However, it is still important to invest in other therapeutic concepts concerning adherence improvement. One goal for ophthalmologists and other healthcare personnel should be to find out about the patients’ requirements of medication management. By asking a set of questions concerning medication management behavior, one could individualize recommendations for an eye drop regimen. This is becoming even more important as working and treatment concepts and capacities of ophthalmological care planning have changed. The number of private practice employees has increased, but most of these are part-time employees; the overall numbers of self-employed ophthalmologists has declined [33]. As a consequence, in some surgeries, patients can no longer be clearly assigned to a specific doctor, which influences the individual doctor-patient relationship. With the help of the patterns identified here, a doctor could utilize consultation conversations to find out which type a patient belongs to, thus integrating an individual’s personality and social structures when designing an individual pattern of safe medication together with each patient. Nevertheless, one must take into account that the behavioral patterns of a person are significantly influenced by their environment and various variables. It is necessary to ensure that specific tools and strategies are provided to the patients so that they can perform and implement new long-term behavior. Long-term adherence implies that a routine incorporating health recommendation has been developed [32]. How behavior pertains to patient adherence is described for diverse models, e.g., the information-motivation-behavioral skills (IMB) model [34] or the social behavior model [35]. Glaucoma, as a chronic disease, requires life-long medication. However, it is not the only disease whose progression is related to poor adherence; it is comparable to that of oral medication for other chronic asymptomatic conditions, like hypertension [36]. Therefore, one could imagine that the results of this study and the patterns identified might also be used to increase adherence of other asymptomatic chronic diseases.

## Figures and Tables

**Table 1:** Interview questionnaire. The interview followed a narrative approach, whereby patients first talked about their disease journey to diagnosis and about their glaucoma in general. This was followed by key questions about adherence and eye drop management, the doctor-patient relationship, and other topics. Additionally, patients had the opportunity to talk about their recommendations for other patients.

1. Narrative stimulus “When was your glaucoma diagnosed and how do you experience it in your daily life? Please tell me everything that comes to mind.”
2. Eye drop management “How about the eye drops that you take, please tell me about that.”
3. Situations leading to non-adherence “Please recall a situation that led you to not using drops. Can you tell me about it?”
4. Doctors visit“Remember the last time you went to the eye doctor and got your prescription for eye drops. Please tell me about it.”
5. Recommendations for other patients “With your experience with glaucoma, what would you recommend to other patients?”
6. Summary question “Do you associate anything else with “drops and glaucoma” that you haven’t spoken of?”

**Table 2:** Glaucoma patients interviewed, according to sex and age.

**Sex**	**Number**
female	12
male	9
**Age [years]**	**Number**
55–70	8
71–80	9
81–90	4
mean age ± SD	73.1 ± 8.9

## Data Availability

Data are available from the corresponding author, upon reasonable request.
